# Best Evidence Summary for Perioperative Pain Management in Patients With Pectus Excavatum

**DOI:** 10.1155/prm/8823617

**Published:** 2025-08-30

**Authors:** Yi Liang, Huajian Peng, Xinxin Huang, Guanbiao Liang

**Affiliations:** Department of Thoracic and Cardiovascular Surgery, The First Affiliated Hospital of Guangxi Medical University, Nanning, China

**Keywords:** evidence-based practices, multimodal analgesia, patient-centered care, pectus excavatum, perioperative pain management

## Abstract

**Background:** Pectus excavatum is a common congenital chest wall deformity that can lead to significant cardiopulmonary compression and psychological distress. The minimally invasive Nuss procedure is the standard treatment, but it often results in severe postoperative pain. Effective perioperative pain management is essential to enhance recovery and improve patient outcomes.

**Objectives:** This study aimed to synthesize the most effective evidence on perioperative pain management in patients with pectus excavatum and to provide evidence-based management methods for clinical teams and patients undergoing this surgery.

**Methods:** Guided by the “6S” pyramid model, we retrieved evidence on perioperative pain management from relevant websites, databases, and unpublished gray literature. The search timeframe ranged from 2014 to December 2024. Two researchers independently evaluated the literature quality using the Appraisal of Guidelines for Research and Evaluation II (AGREE II) for guidelines and the Joanna Briggs Institute (JBI) critical appraisal tool for other types of literature. Two researchers independently extracted and summarized the evidence according to the principle of high-quality evidence and newly published evidence.

**Results:** A total of 39 articles were retrieved, of which 6 were guidelines, 6 were expert consensus, 7 were systematic reviews, 1 was a clinical decision, 11 were randomized controlled trials, and 8 were cohort studies. Overall, 35 pieces of evidence from seven dimensions—general principles, education and counseling, pain assessment, preemptive analgesia, intraoperative analgesia, postoperative pain management, and pain management after discharge—were summarized.

**Conclusions:** This study summarized the best evidence on perioperative pain management in patients with pectus excavatum, providing a comprehensive and scientific approach to enhance recovery and patient satisfaction.

## 1. Introduction

Pectus excavatum (PE), the most common congenital chest wall deformity with an incidence rate of 0.1%–2% [1–3], can lead to cardiac and pulmonary compression, resulting in reduced lung capacity, myocardial damage, and psychological impacts due to the abnormal chest appearance. Early surgical intervention is therefore recommended. The minimally invasive repair of pectus excavatum (MIRPE), also known as the Nuss procedure, introduced by American physician Nuss in 1998, has revolutionized the treatment of PE [4]. This procedure involves inserting one or two convex steel plates under the sternum to reshape the chest wall, which are retained for 2–4 years before removal once the chest wall has set. The Nuss procedure is increasingly accepted due to its minimal trauma, short surgery time, and minimal bleeding [5]. However, the forced outward displacement of the sternum by the steel plates, coupled with the traction and stripping of intercostal muscles during surgery, often results in high visual analog scale (VAS) pain scores of 7–8 in the immediate postoperative period [6]. These scores typically decrease over time, falling below 4 by the 4th to 5th postoperative day [7]. Older patients with less chest wall malleability experience more severe and prolonged pain [8].

Intense postoperative pain can lead to sleep disturbances, fear of deep breathing, coughing, and physical activity, potentially causing pulmonary complications [7, 9]. In severe cases, severe pain can result in long-term protective postures, leading to spinal scoliosis, plate displacement, and surgical failure [10]. Furthermore, if pain is not effectively managed in the initial phase, acute pain may develop into chronic pain, delaying recovery, reducing quality of life, and increasing the burden on patients and their families [11]. Therefore, safe and effective perioperative pain management is crucial for alleviating postoperative pain in Nuss surgery patients, promoting early recovery, and improving clinical outcomes.

The management of postoperative pain in PE surgery is complex and requires a multimodal approach. Historically, thoracic epidural analgesia has been a standard method for managing pain in the early postoperative period after PE repair [12, 13]. However, even with epidural analgesia, severe pain can persist postremoval of the catheter, highlighting the need for additional pain management strategies [14, 15]. Recent studies have explored the use of cryoanalgesia [16–18], where intercostal nerves are targeted with cryoablation during surgery, to enhance recovery and reduce the length of hospital stay. This technique has shown promise in reducing opioid consumption and hospital stay without altering pain scores, suggesting its potential role in a multimodal pain management plan [19, 20]. Moreover, the integration of regional nerve blocks, such as the serratus anterior plane block [21–23], has emerged as a novel strategy for postoperative pain control in MIRPE. A meta-analysis [24] of randomized controlled trials has demonstrated the efficacy of this block in providing analgesia after thoracic surgery, indicating its potential benefits in PE surgery as well. The evolution of pain management strategies in enhanced recovery after surgery (ERAS) pathways has also been significant, with a shift toward multimodal analgesia to improve postoperative outcomes [25–27]. This approach, which includes a combination of different analgesic techniques and medications, aims to minimize the side effects and maximize the benefits of pain relief. The incorporation of personalized pain medicine, including clinical pharmacogenomic testing and patient-reported outcome measurements, is speculated to be the next step in improving perioperative pain management [28].

Overall, the landscape of pain management following PE surgery is rapidly advancing, with new techniques and strategies being developed to improve patient outcomes. The combination of regional nerve blocks, cryoanalgesia, and multimodal analgesia offers a promising approach to managing the intense postoperative pain associated with the Nuss procedure, facilitating earlier recovery and better patient satisfaction [27, 29]. As research continues to evolve, the integration of these evidence-based practices will be crucial in optimizing pain management for patients undergoing PE surgery.

Currently, there is a noticeable absence of targeted, systematic evidence summaries for pain management in patients undergoing PE surgery. This study seeks to address this gap by providing a comprehensive summary of pain management measures tailored to PE surgery. The aim is to deliver an evidence-based reference to inform clinical practice, thereby reducing postoperative pain, accelerating recovery, and improving clinical outcomes.

## 2. Methodology

### 2.1. Problem Establishment

This study employed the PIPOST model to transform clinical issues into evidence-based questions. The target population (P) consisted of patients undergoing MIRPE. The intervention (I) of interest was perioperative pain management. The professionals (P) responsible for applying the evidence included the designated nurses, attending physicians, and patient caregivers. The outcomes (O) evaluated in this study encompassed patient-reported pain scores, length of hospital stay, duration of postoperative chronic pain, quality of life, and the awareness and implementation of pain management strategies by healthcare providers. The setting (S) for evidence application included both hospital wards and home care environments. Lastly, the types of evidence (T) considered in this study ranged from guidelines and best practices to evidence summaries, systematic reviews, expert consensus statements, and original research types.

### 2.2. Evidence Retrieval

This study's literature search strategy adhered to the “6S” evidence pyramid model, conducting a layered search from top-tier to broader sources. We searched Chinese databases including China National Knowledge Infrastructure (CNKI), Wanfang Database, and the China Biomedical Literature Database (SinoMed), alongside English-language resources such as UpToDate, BMJ Clinical Evidence, The Cochrane Library, Joanna Briggs Institute (JBI), Registered Nurses' Association of Ontario (RNAO), National Guideline Clearinghouse (NGC), Guidelines International Network (GIN), National institute for Health and Care Excellence (NICE), Royal College of Anaesthetists EBSCOhost, ProQuest, PubMed, Embase, and Web of Science. We searched for literature on “pectus excavatum/funnel chest/thoracic deformity/sunken chest,” “perioperative period/perioperative/postoperative/intraoperative,” and “pain intervention/pain management/pain evaluation/pain measure,” from January 2014 to December 2024, using a structured approach with subject terms, free-text queries, and Boolean operators to optimize search efficiency. An example of an English database search using PubMed with the corresponding search strategy is shown in Supporting Figure 1. In addition, our search strategy included exploring unpublished gray literature through search engines such as Baidu Scholar.

### 2.3. Inclusion and Exclusion Criteria for Evidence

Inclusion criteria for this study were as follows: (1) Study subjects were patients undergoing MIRPE; (2) literature focused on pain management before, during, and after surgery; (3) literature types were clinical guidelines, summary of evidence, best practice information manual, expert consensus, systematic review, large cohort study, and randomized controlled trial; and (4) language limited to Chinese or English. Exclusion criteria were as follows: (1) repeated publication, incomplete information, unable to obtain the full text of the literature; (2) the document types are plan, draft, report, and abstract; and (3) studies that did not pass the literature quality evaluation.

### 2.4. Literature Screening

The literature retrieved was imported into NoteExpress, where duplicate entries were removed. Two researchers, both trained in evidence-based medicine, conducted independent screenings of the literature. Initial screening involved reviewing titles, abstracts, and keywords. Subsequently, full-text articles were examined and rescreened, with a quality assessment performed on the rescreened literature [30]. In cases where there was disagreement regarding the inclusion of a particular study, a third specialist in the critical field of evidence-based care was consulted to make a final determination on its inclusion status.

### 2.5. Quality Evaluation of the Literature

The quality of guidelines was specifically assessed using the Appraisal of Guidelines for Research and Evaluation II (AGREE II) tool [31], which evaluates the methodological stringency and transparency across six domains: scope and purpose, stakeholder involvement, rigor of development, clarity of presentation, applicability, and editorial independence. Each item is rated on a scale of 1–7, with a higher score indicating higher quality. The score for each area was calculated as follows: (actual score-lowest possible score)/(maximum possible score-lowest possible score). If the scores for six areas are ≥ 60%, the guide is rated as Grade A and can be directly recommended. If one or more areas have scores < 60% and three or more areas have scores ≥ 30%, the guideline is rated as Grade B and can be recommended after revision. If three or more areas have scores < 30%, the guideline is rated as Grade C and is not recommended. The evaluation of systematic reviews is conducted using the JBI systematic review tool [32], which comprises 11 assessment items with options including “Yes,” “No,” “Unclear,” and “Not Applicable”; the assessment of randomized controlled trials is performed with the JBI critical appraisal tool [33] for risk of bias, focusing on internal, external, and statistical conclusion validity; the evaluation of expert opinions is guided by the JBI checklist for textual evidence [34], assessing clarity of opinion sources, expertise, population focus, logical argumentation, literature referencing, and defense of incongruence with sources; and the appraisal of cohort studies is based on the JBI checklist for cohort studies, examining group similarity, exposure measurement, identification and management of confounding factors, outcome measurement at study onset, and follow-up completeness, all of which are developed by JBI to enhance the rigor of evidence synthesis in healthcare decision-making [35]. The evaluation was conducted independently by two reviewers, with a third party involved to resolve discrepancies, ensuring that only the most robust evidence was synthesized, thereby enhancing the credibility of our research findings.

### 2.6. Evidence Extraction and Summary

Two researchers independently abstracted data from the included studies, encompassing details such as literature titles, authors, sources, evidence types, publication dates, references, and the strength and level of evidence-based recommendations. This extraction process was followed by cross-verification between the two researchers, with discrepancies resolved by a third researcher. The three researchers collaboratively synthesized the evidence, prioritizing divergent conclusions and favoring high-quality, recent, and authoritative sources in cases of conflict.

Employing the JBI evidence grading system [36, 37], evidence was ranked across five levels, with Level 1 being the most robust and Level 5 the least. Concurrently, the FAME framework of JBI, which evaluates the validity, applicability, meaningfulness, and effectiveness of evidence, guided the research team in classifying the evidence recommendations as either Grade A (strong recommendation) or Grade B (weak recommendation). This systematic approach ensured a rigorous and consistent evaluation of the evidence.

## 3. Results

### 3.1. Search Results

**Figure 1:**
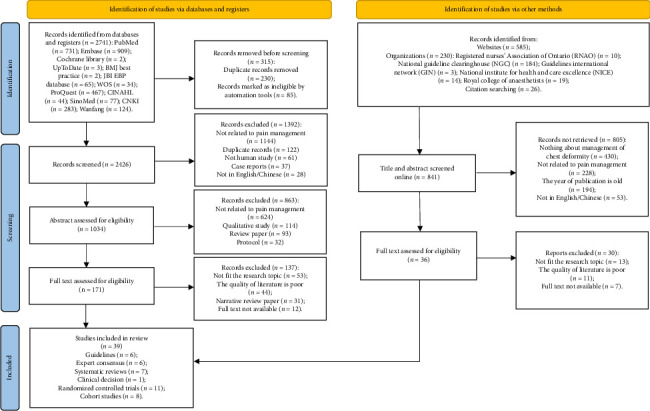
The literature screening process.

The initial search for this study yielded a total of 2741 articles from databases and registers, complemented by an additional 841 articles sourced through other methods. Upon reviewing the titles and abstracts, 207 articles were selected for full-text screening. After a thorough evaluation, 39 articles were incorporated into the study, which included six guidelines [38–43], six expert consensus [44–49], seven systematic reviews [50–56], one clinical decision [57], 11 randomized controlled trials [15, 58–67], and eight cohort studies [68–75]. The literature screening process is shown in Figure 1. The general characteristics of the included literature are shown in Supporting Table 1.

### 3.2. Quality Evaluation Results of the Included Literature

#### 3.2.1. Quality Evaluation Results of Guidelines

This study incorporated six clinical guidelines [38–43], with evaluation results detailed in Supporting Table 2. The guidelines' overall quality was rated at 5.96 using the AGREE II tool. Four of these guidelines achieved a score of 60% or higher across all domains [38–40, 43], earning an A-level recommendation, whereas the remaining two were categorized as B-level [41, 42]. The average scores across the six dimensions were as follows: scope and purpose (93.06%), stakeholder involvement (83.34%), rigor of development (82.03%), clarity of presentation (94.21%), applicability (60.24%), and editorial independence (92.02%). Consequently, these guidelines were deemed to be of high quality and were included in the summary.

#### 3.2.2. Quality Evaluation Results of Expert Consensus

Six expert consensus articles [44–49] were reviewed, and their evaluation findings are presented in Supporting Table 3. The articles [45, 46, 48, 49] each received affirmative responses for five of the six evaluation criteria, with the sixth criterion—“Is any incongruence with the literature/sources logically defended?”—being marked as “unclear.” The remaining two expert consensus articles [44, 47] responded affirmatively to all evaluation items. Given these results, all six expert consensus articles were considered high quality and included in the study.

#### 3.2.3. Quality Evaluation Results of Systematic Reviews

This study included seven systematic reviews [50–56], all published within the last 5 years. Out of these seven reviews, five concentrated specifically on pain management strategies following MIRPE [51, 52, 54–56]. The remaining two reviews [50, 53], while broadening their scope to encompass a range of pediatric chest surgeries, still included our focal topic—PE surgery. Consequently, all seven were considered in the quality evaluation as depicted in Supporting Table 4. It can be observed that these studies generally performed well in clearly stating the research question, setting appropriate inclusion criteria, employing suitable search strategies, utilizing adequate search resources, establishing standards for appraising studies, and conducting double independent reviews. Despite some deficiencies in minimizing errors in data extraction and assessing the potential for publication bias, the majority of the reviews were able to provide data support for policies and practices and offer guidance for future research. Collectively, these systematic reviews have provided valuable insights and recommendations for pain control methods following MIRPE and were included in the summary.

#### 3.2.4. Quality Evaluation Results of Randomized Controlled Trials

Eleven randomized controlled trials [15, 58–67] were included in this study, and the quality assessment results are presented in Supporting Table 5. All studies utilized true randomization methods for participant grouping and maintained comparable treatment groups at baseline. Except for two studies [15, 64], the remaining achieved blinding of participants and outcome assessors to reduce performance and detection biases. All studies treated the intervention groups identically except for the intervention of interest and measured outcomes in a consistent and reliable manner. In terms of participant retention, all studies completed follow-ups and adequately described and analyzed differences in follow-up. Furthermore, all studies analyzed participants in the groups to which they were randomized and employed appropriate statistical methods. Although a few studies had unclear aspects regarding allocation concealment [15, 58] and blinding of some treatment providers and outcome assessors [15, 64, 65]; overall, these studies were of high quality, providing a solid foundation for evidence-based practice in pain control methods following MIRPE, and were included in the summary.

#### 3.2.5. Quality Evaluation Results of Cohort Studies

Eight cohort studies [68–75] were included in this research. As shown in Supporting Table 6, the eight cohort studies demonstrated high-quality standards, ensuring similarity between study and control groups at baseline and consistent measurement of exposure. They effectively recognized and addressed confounding variables, reliably measured outcomes, and reported adequate follow-up times, offering solid evidence for pain management post-PE repair, and were included in the overall summary.

#### 3.2.6. Quality Evaluation Results of Clinical Decision

One clinical decision [57] from UpToDate was included in the final set of literature, which focuses on the treatment methods for PE. This article adhered to rigorous evidence development processes and standards. Upon tracing the original literature, we identified one randomized controlled trial [65] and four cohort studies [71–73, 75], all pertaining to anesthesia and analgesia in PE surgery. We conducted a comprehensive quality assessment of these five primary studies, as presented in Supporting Tables 5 and 6, to ensure the reliability of our findings.

### 3.3. Summary and Description of Evidence

In total, 35 pieces of evidence from seven dimensions (general principles, education and counseling, pain assessment, preemptive analgesia, intraoperative analgesia, postoperative pain management, and pain management after discharge) were extracted and summarized. The details are presented in Table 1.

## 4. Discussion

### 4.1. General Principles

Evidence items 1 to 5 highlight the critical principles of perioperative pain management for PE patients, emphasizing the multifaceted nature of this process. These principles underscore the importance of a comprehensive approach that includes a multidisciplinary team, goal-oriented strategies, patient-centered care, standardized pain assessment, and multimodal analgesia.

The multidisciplinary team's role in perioperative pain management for PE surgery extends beyond the immediate surgical team to include a network of professionals who contribute to the patient's journey from preoperative assessment through to postoperative recovery. This collaborative model is reflective of the evolving landscape of pain management, where traditional acute pain services are expanding their remit to encompass the entire patient pathway. By integrating the expertise of thoracic surgeons, anesthesiologists, nurses, physical therapists, and psychologists, among others, the team can address the complex interplay of physical, psychological, and rehabilitative needs that are integral to effective pain management [40, 43, 44, 47, 49]. This holistic approach ensures that each facet of the patient's experience is considered, from the initial preparation for surgery to the long-term recovery process, thereby optimizing outcomes and enhancing the quality of care [76–78].

Furthermore, the goal-oriented strategy is not just about achieving immediate pain relief but also about setting the stage for a swift and uncomplicated recovery [44, 49]. By focusing on specific milestones, such as the ability to breathe deeply and cough effectively, the team can measure progress and adjust treatments as needed, ensuring that each step toward recovery is supported by effective pain control [46, 47]. The empowerment of patients and their caregivers through patient-centered care is a significant shift from a paternalistic model of healthcare delivery. This approach recognizes the patient as a partner in their care, which can lead to more informed decisions and a greater sense of control over their recovery [40]. This is particularly important in the context of PE, where the psychological impact of the condition and the surgery itself can be significant [79, 80]. By involving patients in the decision-making process, healthcare providers can better address the patient's concerns and expectations, leading to a more personalized and effective pain management plan [43, 44, 47, 49].

Moreover, standardized pain assessment tools are vital for monitoring pain levels and guiding treatment adjustments, providing a consistent framework for evaluating the effectiveness of pain management strategies and identifying any complications early [39, 48, 49]. The promotion of multimodal analgesia reflects a contemporary approach to pain relief that leverages the combined effects of various medications and techniques [42–47, 49]. This strategy acknowledges the diverse mechanisms of pain and the individual responses to various therapies, aiming to optimize pain relief while minimizing side effects [38–40].

### 4.2. Education and Counseling

Evidence items 6, 7, 8, and 9 highlight the importance of education and counseling in the comprehensive management of perioperative pain for patients with PE. Preoperative education is crucial for setting the stage for patients and their caregivers, providing them with an understanding of what to expect during and after surgery, including the nature of the procedure, the types of pain they may experience, and the strategies that will be employed to manage it [44, 47]. This preparation helps to alleviate preoperative anxiety and improves adherence to the prescribed pain management regimen, which is essential for achieving better pain outcomes [40, 43, 74]. While adult education focuses on self-management of complex analgesic regimens and chronic pain risks, pediatric counseling prioritizes caregiver empowerment and age-appropriate pain communication tools to reduce perioperative distress.

Moreover, shared decision-making is another critical component of patient-centered care, allowing patients and their caregivers to be actively involved in the selection of pain management approaches [40, 43, 44]. This collaborative process not only offers psychological benefits by reducing anxiety but also fosters a sense of control and involvement in one's own care, which can lead to increased satisfaction with the treatment process [47]. By engaging in shared decision-making, patients are more likely to feel empowered and invested in their recovery, which can positively impact their pain management experience [81].

Also, postoperative recovery guidance emphasizes the significance of early mobilization and adherence to physical therapy and rehabilitation plans. These components are integral to the recovery process and are known to contribute positively to pain control and overall healing. By understanding the importance of these activities, patients are more likely to engage in them, which can lead to improved outcomes and a more effective management of postoperative pain [40, 49].

Finally, information delivery, both verbally and in writing, ensures that patients fully understand their pain management plan [43, 44, 47, 49]. This dual approach to communication is crucial for patient reference and understanding, promoting a deeper comprehension of their treatment and facilitating better self-management of postoperative pain. With clear and accessible information, patients can make more informed decisions about their care and engage more effectively in their pain management, leading to a more positive surgical experience and recovery [49].

### 4.3. Pain Assessment

Pain assessment, as delineated by evidence items 10 through 14, is a cornerstone in the management of perioperative pain for patients with PE. Employing multidimensional tools facilitates a comprehensive evaluation of both the numerical and functional aspects of pain, which is pivotal for guiding treatment and ensuring effective management [43, 47–49]. Unidimensional measures, focusing solely on pain intensity, often fail to account for other contributing factors to the pain experience, such as physical function, emotional distress, sleep impact, and past pain treatments [82–84]. The American Pain Society (APS) has emphasized the importance of considering these elements and has recommended the use of validated pain assessment tools that can monitor patients' pain and track progress in postoperative pain levels [43]. These tools often assess the impact of pain on function and sleep, leading to more tailored pain care plans in the perianesthesia setting. For instance, the Clinically Aligned Pain Assessment Tool (CAPA) encourages a structured conversation with patients about their pain, covering aspects such as pain intensity, sleep, functioning, comfort, change in pain, and pain control [85]. The use of such tools can improve patient satisfaction with pain care and enhance nurses' ability to make informed clinical decisions.

The preoperative risk assessment is instrumental in identifying factors that may influence postoperative pain, such as the severity of the deformity, psychological status, and lifestyle habits [43, 47–49]. This process allows for a tailored approach to pain management, potentially enhancing outcomes and minimizing complications. Meanwhile, continuous monitoring of pain trajectories by tracking individual pain scores is essential for early identification of complications and for adjusting treatment strategies, thereby maintaining the effectiveness of pain management [40, 43, 46, 48, 49]. Considering the impact of mood and sleep on pain reporting is crucial for the accurate interpretation of pain scores. This contextualization allows healthcare providers to adjust treatment plans to more accurately address the patient's actual pain experience [40, 48, 49].

Furthermore, adjusting assessment methods for special populations is crucial, as not all patients can effectively communicate their pain due to communication disorders or other limitations [40, 48, 49]. Observational scales are the most common instruments to evaluate pain in individuals with communication disorders, followed by physiological measures and facial recognition systems [86]. While adults with cognitive impairments may benefit from adapted versions of standard scales (e.g., simplified NRS) combined with caregiver input, pediatric populations require fundamentally different tools. In children, specialized pain assessment scales like the Face, Legs, Activity, Cry, and Consolability (FLACC) scale focus exclusively on observable behaviors and physiological responses87, with modifications for developmental stages (e.g., FLACC-R for cognitively impaired children) [87]. Such tools are designed to be sensitive to the unique needs of these populations, ensuring that pain assessment is accurate and appropriate [88].

### 4.4. Preemptive and Intraoperative Analgesia

Evidence items 15 through 19 provide a comprehensive overview of preemptive and intraoperative analgesia strategies in PE surgery to minimize postoperative pain and enhance recovery. Preemptive analgesia, as suggested by evidence item 15, involves the use of nonsteroidal anti-inflammatory drugs (NSAIDs) such as flurbiprofen axetil and parecoxib before surgery, which can reduce postoperative pain and the need for opioids [39, 40, 49, 53, 75]. This strategy is supported by evidence that demonstrates the benefits of NSAIDs in reducing inflammation and pain, without the risk of respiratory depression associated with opioids [53, 75].

A multimodal approach to intraoperative analgesia is essential, as it has been shown to improve postoperative pain and functional outcomes, reduce postoperative opioid requirements and related adverse effects, and decrease complications [39, 43]. The choice of analgesic agents must consider patient-specific factors, including characteristics, comorbidities, pre-existing analgesic use, potential drug interactions, and the nature of the surgery [89]. This strategy involves both systemic and regional/local analgesia, which may be supplemented by nonpharmacological techniques in the postoperative phase. There is substantial evidence supporting the effectiveness of medications such as paracetamol, NSAIDs, NMDA antagonists, steroids, and α-2 adrenergic agonists, which are recommended for their analgesic properties and ability to reduce opioid consumption [53, 56, 58, 62].

Procedure-specific regional anesthesia is a pivotal part of the multimodal analgesia strategy for PE surgery [38, 57], as emphasized in evidence item 18. This method targets the nerves directly affected by the surgery, enhancing pain relief and minimizing the need for systemic opioids [12]. Techniques such as thoracic epidural blocks [15], thoracic paravertebral blocks [51, 53, 67], erector spinae plane blocks [51, 53, 60, 61], and intercostal nerve blocks [63, 66] are utilized to manage postoperative pain by administering local anesthetics near the chest wall nerves, thereby blocking pain signals. However, these methods demand technical proficiency and carry risks like pneumothorax or nerve injury. Cryoablation [50, 52, 55] stands out as an innovative regional anesthesia approach. This procedure applies a cryoprobe to the intercostal nerves, which innervate the chest wall, cooling them to a temperature that induces temporary nerve damage and blocks pain signals. Cryoablation has been linked to reduced hospital stays, decreased opioid usage, and a lower incidence of both short-term and long-term complications [69, 71, 72]. As a nonpharmacological approach, it enhances recovery quality and aligns with the ERAS protocol, offering a valuable pain management option for patients undergoing PE repair [68, 70, 73–75].

Evidence item 19 highlights a critical aspect of surgical technique in PE repair: the significance of gentle tissue manipulation. This principle is based on the well-established concept that reducing surgical trauma can lead to decreased postoperative pain and accelerated recovery [40, 49]. By employing gentle tissue handling, surgeons can minimize the disruption of tissues, which in turn can reduce inflammation and the body's stress response to injury [90].

### 4.5. Postoperative Pain Management

Postoperative pain management for PE patients is specifically tailored to address the unique challenges and needs of this patient population. The following measures, as outlined in evidence 20 to 29, are designed to minimize pain, promote comfort, and facilitate recovery while reducing the risks associated with postoperative complications and delayed discharge.

Multimodal analgesia is the cornerstone of postoperative pain control for PE patients, which includes a combination of medications and techniques to optimize pain relief and minimize side effects [39, 43, 44]. Simple analgesics such as paracetamol and NSAIDs are often the first line of treatment, with opioids being used when necessary for acute pain management [42, 45]. Immediate-release opioids are preferred for their rapid and titratable analgesic effects over modified-release formulations, which have been associated with increased adverse events and persistent postoperative opioid use [42, 44]. The oral route is preferred, and the lowest effective dose should be given, with sedation score monitoring for early detection of opioid-induced ventilatory impairment [42, 44].

As recommended in evidence item 25, patient-controlled analgesia (PCA) is a preferred method for postoperative pain management in PE surgery. It allows patients to self-administer medication promptly when they experience pain, providing a personalized approach to pain relief [45]. This strategy is particularly effective in the immediate postoperative period, reducing basal pain and enhancing patient satisfaction by giving them control over their comfort [54, 64]. PCA also supports a multimodal analgesic regimen, contributing to a comprehensive pain management plan for PE patients [91, 92].

Regional anesthesia, particularly thoracic epidural analgesia and paravertebral blocks, has been shown to be highly effective in controlling postoperative pain in PE patients by directly targeting the nerve pathways involved in pain transmission from the surgical site [44, 51, 56]. This approach not only provides superior pain relief but also reduces the reliance on opioids, thereby minimizing the risk of opioid-related side effects, such as respiratory depression and constipation [51, 56]. Nonpharmacological analgesia complements pharmacological methods by addressing the multifaceted nature of postoperative pain [40, 41, 43, 49]. Physical therapies like cryotherapy and thermotherapy, integrative treatments such as massage and acupuncture, and psychological interventions, including cognitive–behavioral therapy, are valuable in managing pain by targeting its physical, emotional, and psychological aspects [93–96].

The reassessment of pain after intervention, as emphasized in evidence 28, is a critical step in the dynamic process of postoperative pain management for PE patients [44]. This practice ensures that any changes in the patient's pain status are promptly identified and addressed, allowing for the adjustment of treatment plans to maintain or improve analgesic efficacy [97, 98]. Regular pain reassessments can help to evaluate the effectiveness of the current analgesic regimen, identify any potential complications or side effects from pain management strategies, and tailor the treatment to the individual patient's needs [99, 100]. This iterative process of assessment and adjustment is particularly important in the context of PE surgery, where pain can be intense and variable, and where effective pain control is crucial for promoting early mobilization and recovery [101].

Furthermore, evidence 29 also highlights the importance of involving advanced pain management teams in the care of PE patients whose pain does not follow the expected trajectory or who require advanced analgesic techniques [44]. These teams, often composed of anesthesiologists, pain specialists, nurses, and other healthcare professionals, possess specialized knowledge and skills in managing complex pain cases. Their involvement can lead to the implementation of more sophisticated and targeted pain management strategies, such as neuroaxial analgesia, implantable devices, or interventional procedures, which may not be part of the standard postoperative pain management protocol [40, 44]. Advanced pain management teams can also provide additional support and education to patients and their families, helping to manage expectations and enhance the overall quality of care [102, 103].

It is worth noting that postoperative pain management strategies diverge between adults and pediatric patients in both pharmacological and nonpharmacological approaches. Adults often benefit from advanced regional techniques (e.g., thoracic epidural analgesia) and opioid-sparing multimodal regimens to address prolonged pain trajectories, while pediatric care emphasizes weight-based opioid dosing, NSAID-first protocols, and developmentally tailored interventions (e.g., distraction therapy) to minimize psychological trauma and respiratory risks. Cryoablation has emerged as particularly impactful for children undergoing Nuss procedures, reducing both opioid dependence and hospital stays, whereas adults may require longer term pain service involvement to prevent chronic postsurgical pain.

### 4.6. Pain Management After Discharge

Postoperative pain management after discharge is a critical yet often overlooked phase in the recovery process for patients with PE. This period is particularly vulnerable as patients transition from the structured hospital environment to home care, where they must manage their pain with limited professional supervision. The challenge is to ensure that patients receive adequate pain relief while minimizing the risk of opioid overuse, misuse, and the development of chronic postsurgical pain [44]. Evidence items 30 to 35 highlight the importance of a structured approach to postdischarge pain management to ensure continuity of care and to minimize the risk of chronic pain and opioid dependency.

Evidence 30 highlights the importance of establishing a local protocol for prescribing discharge medications for a limited duration, specifically no more than 7 days [40, 42, 43, 49]. This strategy aims to prevent repetitive opioid prescriptions, thereby reducing the risks of dependency and adverse events associated with long-term opioid use. Evidence 31 emphasizes the need for patient education on the proper use of pain medications, including safe storage and disposal of unused drugs, and potential side effects [40, 43, 44, 49]. This education is crucial in empowering patients to manage their pain effectively and to understand the risks associated with their medications.

Evidence 32 suggests the creation of a personalized pain management plan in collaboration with patients, which may include a gradual reduction in opioid use [40, 43, 49]. This approach recognizes the individual variability in pain experiences and treatment responses, promoting a tailored strategy for pain management. Evidence 33 calls for the identification of high-risk patients and the development of a multidisciplinary analgesic plan, potentially involving pain specialists. This proactive approach is essential for preventing the progression to chronic pain states and ensuring that patients receive the appropriate level of care [40, 43, 44, 49].

Evidence 34 focuses on preventing repetitive opioid prescriptions in the community and refers back to the surgical team for unmanageable postoperative pain. This community-based approach ensures that patients receive continuous care and are not left to manage severe pain without professional support [40, 43, 44, 49]. Evidence 35 stresses the importance of regular follow-ups, including face-to-face pain assessments, to adjust pain management plans as needed. These follow-ups are vital for identifying patients at risk of developing chronic postsurgical pain and for optimizing treatment plans to provide effective pain relief [40, 43, 49].

Moreover, postdischarge pain management requires distinct considerations for adult and pediatric populations. Adults often need structured opioid tapering protocols and multidisciplinary follow-up to mitigate chronic pain risks, while pediatric care focuses on caregiver education about age-appropriate medication dosing and nonpharmacological comfort measures. Children typically require shorter duration opioid prescriptions (3–5 days) with strict parental supervision, whereas adults may benefit from longer term pain service coordination, particularly those with pre-existing pain conditions. Both groups benefit from scheduled follow-ups, but pediatric plans must additionally address developmental milestones and family-centered recovery support.

### 4.7. Limitation

Limitations inherent in the evidence base for perioperative pain management in patients with PE arise from the sheer volume and broad scope of the included studies, which introduce considerable heterogeneity and complicate the synthesis and application of the findings. To operationalize this evidence into clinical practice, it is essential for healthcare providers to take into account the specific clinical context and the individual needs and preferences of patients when formulating a comprehensive pain management plan. Additionally, the international origin of the literature included in this study poses challenges for its direct application in different cultural and healthcare settings. When adapting evidence for use in China, for example, it is crucial to select evidence that is relevant and reflective of the local cultural background, disease characteristics, and treatment methods. This selective approach is vital for developing intervention strategies that are not only culturally sensitive but also tailored to the unique needs of Chinese patients with PE, with the goal of effectively mitigating their symptoms and improving their long-term quality of life.

## 5. Conclusion

The best evidence for perioperative pain management in patients with PE points toward a multidisciplinary, goal-oriented, patient-centered, and multimodal approach. The synthesis of evidence from various sources underscores the importance of a comprehensive strategy that includes preoperative education, standardized pain assessment, and a combination of pharmacological and nonpharmacological interventions. The use of regional anesthesia, such as thoracic epidural analgesia and intercostal nerve blocks, along with cryoanalgesia, has shown promise in reducing opioid consumption and hospital stay without altering pain scores, suggesting its potential role in a multimodal pain management plan. The integration of personalized pain medicine, including clinical pharmacogenomic testing and patient-reported outcome measurements, is speculated to be the next step in improving perioperative pain management.

For clinical practice, these findings emphasize the need for healthcare providers to adopt a patient-centered approach, involving patients in decision-making processes and tailoring pain management plans to individual needs. The implications for future research include the need for further investigation into the long-term effects of different pain management strategies, the development of personalized pain management protocols, and the evaluation of the cost-effectiveness of various analgesic techniques. As the landscape of pain management following PE surgery continues to evolve, the integration of these evidence-based practices will be crucial in optimizing pain management for patients undergoing PE surgery.

## Figures and Tables

**Table 1:** Summary of evidences for perioperative pain management in patients with pectus excavatum.

Evidence subject	Evidence content	Evidence level	Recommended level
General principles	1. Multidisciplinary: a collaborative approach among healthcare professionals, including thoracic surgeons, anesthesiologists, nurses, physical therapists, and psychologists, is crucial for delivering a comprehensive and effective perioperative pain management for patients with pectus excavatum [40, 43, 44, 47, 49].	Level 5b	A
	2. Goal-oriented: to ensure safe, effective, and continuous pain control, minimize side effects, optimize physical and psychological recovery, enhance patient satisfaction, and reduce postoperative complications and pain-related issues [44, 46, 47, 49].	Level 5b	A
	3. Patient-centered: empowering patients and involving their caregivers in the decision-making process to ensure personalized pain management [40, 43, 44, 47, 49].	Level 5b	A
	4. Guided by pain assessment: pain management is customized based on ongoing evaluation using standardized tools to monitor and adjust treatment effectively [39, 48, 49].	Level 5b	A
	5. Multimodal analgesia encouraged: utilizing a combination of medications and techniques to optimize pain relief and minimize side effects, enhancing patient recovery [38–40, 42–47, 49].	Level 1b	A

Education and counseling	6. Preoperative education: prepare patients and caregivers with information on surgical procedure, postoperative pain, management strategies, and recovery expectations [40, 43, 44, 47, 74].	Level 3c	A
	7. Shared decision-making: engage patients and caregivers in selecting pain management approaches, offering psychological support to reduce anxiety and increase involvement in care [40, 43, 44, 47].	Level 5b	A
	8. Postoperative recovery guidance: provide guidance on postoperative activities, emphasizing early mobilization, along with a physical therapy and rehabilitation plan [40, 49].	Level 5b	A
	9. Information delivery: ensure information is communicated both orally and in writing for patient reference and understanding [43, 44, 47, 49].	Level 5b	B

Pain assessment	10. Use multidimensional tools: employ a combination of numerical and functional pain assessment tools to guide treatment [43, 47–49].	Level 5b	A
	11. Preoperative risk assessment: screen pectus excavatum patients for factors influencing postoperative pain, such as the severity of the deformity, psychological status, and lifestyle habits [43, 47–49].	Level 5b	A
	12. Monitor pain trajectories: track individual pain scores to identify complications and adjust treatment accordingly [40, 43, 46, 48, 49].	Level 5b	A
	13. Contextualize pain scores: consider how factors like mood and sleep can affect pain reporting and interpret scores in context [40, 48, 49].	Level 5b	A
	14. Special population adjustments: adapt assessment methods for pectus excavatum patients with special needs, especially for pediatric patients or those with cognitive impairments [40, 48, 49].	Level 5b	A

Preemptive analgesia	15. Preoperative use of NSAIDs: the use of nonsteroidal anti-inflammatory drugs (NSAIDs) is recommended for preemptive analgesia the day before surgery. Examples include flurbiprofen axetil and parecoxib (contraindicated for those allergic to sulfonamides) [39, 40, 49, 53, 75].	Level 1b	B

Intraoperative analgesia	16. Multimodal intraoperative analgesia: intraoperative analgesia should be multimodal and extend into the postoperative period [39, 43].	Level 1a	A
	17. Personalized drug selection: for pectus excavatum surgery, intraoperative analgesia should involve a personalized selection of drugs such as acetaminophen, NSAIDs, NMDA antagonists, steroids, and α-2 agonists, combined with systemic and regional/local anesthetic techniques to optimize pain control [53, 56, 58, 62].	Level 1a	A
	18. Regional anesthesia: procedure-specific regional anesthesia, such as thoracic epidural blocks, thoracic paravertebral blocks, erector spinae plane blocks, intercostal nerve blocks, and cryoablation, should be considered an essential element of multimodal analgesia for pectus excavatum surgery where feasible [15, 38, 50–53, 55, 57, 59–61, 63, 65–75].	Level 1a	A
	19. Gentle manipulation during surgery: the surgeon should manipulate tissues gently during the procedure, plan the surgical field rationally, and avoid unnecessary trauma [40, 49].	Level 5b	A

Postoperative pain management	20. Postoperative multimodal analgesia: to provide a combination of various analgesic medications, techniques, and nonpharmacological interventions for postoperative pain management in both children and adults [39, 43, 44].	Level 1a	A
	21. Controlling basal pain: intravenous NSAIDs or acetaminophen are recommended at appropriate doses throughout the postoperative period to control basal pain [42, 45].	Level 1a	A
	22. Opioids for rapid analgesia: in acute settings, immediate-release opioids are recommended for rapid and adjustable analgesia instead of modified-release or compound formulations [42, 44].	Level 5b	A
	23. Minimizing analgesic doses: use the lowest effective dose of analgesics, including opioids, and prefer oral administration, when possible, with age-appropriate initial dosing [42, 44].	Level 5b	A
	24. Minimizing sedative coadministration: Avoid coadministering sedative medicines; if necessary, use the lowest doses for the shortest duration and monitor for opioid-induced respiratory depression [42, 44].	Level 5b	A
	25. Patient-controlled analgesia: consider using intravenous patient-controlled analgesia (PCA) and ensure proper monitoring is in place to allow patients to self-manage their pain as needed [45, 54, 64].	Level 3a	A
	26. Regional anesthesia: recommend the implementation of regional anesthesia as an essential component of a multimodal analgesic regimen, with appropriate monitoring such as pulse oximetry to provide targeted postoperative pain relief [44, 51, 56].	Level 1a	A
	27. Incorporating nonpharmacological analgesia: integrating physical therapies, integrative treatments, psychological interventions, and neurostimulation methods into pain management plans [40, 41, 43, 49].	Level 5b	A
	28. Reassessment of pain after intervention: emphasizing the need to reassess pain following any intervention to adjust treatment plans accordingly [44].	Level 5b	A
	29. Advanced pain management teams: Involving pain management teams for patients whose pain does not follow the expected trajectory or who require advanced analgesic techniques [44].	Level 5b	A

Pain management after discharge	30. Medication management: establish a local protocol for prescribing discharge medications for no more than 7 days, avoiding repetitive opioid prescriptions to reduce dependency and adverse event risks [40, 42, 43, 49].	Level 5b	A
	31. Patient education: instruct patients on the proper use of pain medications, safe storage and disposal of unused drugs, and potential side effects [40, 43, 44, 49].	Level 5b	A
	32. Individualized pain plan: collaborate with patients to create a personalized pain management plan, including a gradual reduction in opioid use [40, 43, 49].	Level 5b	A
	33. Long-term pain management: identify high-risk patients, develop a multidisciplinary analgesic plan, and involve pain specialists when necessary [40, 43, 44, 49].	Level 5b	A
	34. Community management: prevent repetitive opioid prescriptions in the community and refer back to the surgical team for unmanageable postoperative pain [40, 43, 44, 49].	Level 5b	A
	35. Follow-up: schedule regular follow-ups, conduct face-to-face pain assessments, and adjust pain management plans accordingly [40, 43, 49].	Level 5b	A

## Data Availability

Data sharing is not applicable to this article as no datasets were generated or analyzed during the current study.

## Supporting Information

Supporting Figure 1 shows the comprehensive PubMed search strategy used in this study, which includes relevant keywords and MeSH terms related to pectus excavatum and perioperative pain management. Supporting Table 1 provides an overview of the general characteristics of the 39 included literature, including source, year of publication, type of literature, and specific theme. Supporting Table 2 presents the quality evaluation of the six included guidelines, showing standardized scores in various domains and overall quality grades. Supporting Table 3 evaluates the quality of the six expert consensuses, assessing factors such as the clarity of the opinion source, standing in the field, and logical defense. Supporting Table 4 provides a quality evaluation of the seven systematic reviews, examining aspects such as the clarity of the review question, appropriateness of inclusion criteria, and adequacy of the search strategy. Supporting Table 5 evaluates the quality of the 11 randomized controlled trials, assessing aspects related to selection bias, intervention administration, outcome assessment, and statistical validity. Supporting Table 6 provides a quality evaluation of the eight cohort studies, examining factors such as similarity of groups, exposure measurement, confounding factors, and follow-up completeness.
