# Supplementary material for: Best Evidence Summary for Perioperative Pain Management in Patients With Pectus Excavatum
Source: Pain Res Manag. 2025 Aug 30;2025:8823617. doi: 10.1155/prm/8823617 (PMC12413950; doi:10.1155/prm/8823617)
Supplement: Supporting Information — Additional supporting information can be found online in the Supporting Information section. [file 8823617.f1.docx]

**Best Evidence Summary for Perioperative Pain Management in Patients with Pectus Excavatum**

**Supplementary description**

Supplementary Figure 1 shows that the PubMed search strategy used in this study is comprehensive and well-structured. It includes a combination of relevant keywords and MeSH terms related to pectus excavatum, perioperative pain management, and various pain management techniques. This strategy ensures that all potentially relevant studies are identified, providing a solid foundation for the systematic review and analysis of the literature.

Supplementary Table 1 provides a detailed overview of the general characteristics of the 39 included literatures. It includes information on the source, year of publication, type of literature, and the specific theme of each study. This table helps to understand the scope and diversity of the studies included in this review, highlighting the various guidelines, expert consensuses, systematic reviews, RCTs, and cohort studies that contribute to the evidence base for perioperative pain management in patients with pectus excavatum.

Supplementary Table 2 presents the quality evaluation of the six included guidelines. The table shows the standardized scores in various domains, including scope and purpose, stakeholder involvement, rigour, clarity, applicability, and independence. Each guideline is also assigned an overall quality grade based on these scores. This evaluation helps to assess the reliability and applicability of the guidelines in clinical practice, with most guidelines receiving high scores and an "A" or "B" quality grade.

Supplementary Table 3 evaluates the quality of the six expert consensuses included in this study. It assesses whether the source of the opinion is clearly identified, has standing in the field of expertise, and focuses on the interests of the relevant population. It also examines whether the opinion is logically defended, references the extant literature, and addresses any incongruence with the literature/sources. This evaluation highlights the strengths and potential limitations of each expert consensus in providing guidance for perioperative pain management.

Supplementary Table 4 provides a quality evaluation of the seven systematic reviews included in this study. It examines whether the review question is clearly stated, the inclusion criteria are appropriate, the search strategy is adequate, and the sources and resources used are sufficient. It also assesses whether the criteria for appraising studies are appropriate, critical appraisal is conducted independently, methods to minimize errors in data extraction are used, and the likelihood of publication bias is assessed. Additionally, it evaluates whether recommendations for policy and practice are supported by the data and whether specific directives for new research are appropriate. This evaluation helps to determine the reliability and usefulness of the systematic reviews in informing clinical decision-making.

Supplementary Table 5 evaluates the quality of the 11 randomized controlled trials included in this study. It assesses various aspects related to selection and allocation bias, administration of intervention/exposure, assessment, detection, and measurement of outcomes, as well as participant retention and statistical conclusion validity. This evaluation helps to identify the strengths and potential weaknesses of each RCT in providing robust evidence for the effectiveness of different pain management interventions in patients with pectus excavatum.

Supplementary Table 6 provides a quality evaluation of the eight cohort studies included in this study. It examines whether the two groups were similar and recruited from the same population, the exposures were measured similarly, the exposure was measured in a valid and reliable way, confounding factors were identified and addressed, the groups/participants were free of the outcome at the start of the study, the outcomes were measured in a valid and reliable way, the follow-up time was sufficient, follow-up was complete, and appropriate statistical analysis was used. This evaluation helps to assess the reliability and validity of the cohort studies in providing evidence for the effectiveness of different pain management strategies in a real-world setting.

**#1** (((period, perioperative [MeSH Terms]) OR (postoperative)) OR (perioperative)) OR (after surgery)

**#2** ((((pectus excavatum) OR (funnel chest)) OR (pectus abnormal*)) OR (thoracic deformity)) OR (sunken chest)

**#3** (((pain manage*) OR (pain assess*)) OR (pain evaluat*)) OR (pain measure*)

**#4** #1 AND #2 AND #3

**Supplementary Figure 1.** PubMed search strategy.

| **Supplementary Table 1.** The general characteristics of the included literatures (n = 39). | | | | |
| --- | --- | --- | --- | --- |
| Included literature | Source | Year of publication | Type of literature | The literature theme |
| Dunning et al.^37^ | PubMed | 2024 | Guideline | Pectus care guidelines: joint societies' recommendations for pectus treatment. |
| Vittinghoff et al.^38^ | PubMed/European Society for Pediatric Anesthesiology | 2024 | Guideline | Postoperative pain management in children |
| Royal College of Anesthetists^39^ | Royal College of Anesthetists | 2024 | Guideline | Guidelines for the provision of pediatric anesthesia services |
| Chinese Society of Integrated Traditional and Western Medicine anesthesia professional committee^40^ | WangFang | 2021 | Guideline | Acupoint stimulation assisted treatment of postoperative pain |
| Cravero et al.^41^ | PubMed/ Society for Pediatric Anesthesia | 2019 | Guideline | The use of opioids in children during the perioperative period |
| Chou et al.^42^ | PubMed/ American Pain Society | 2016 | Guideline | Management of postoperative pain |
| El-Boghdadly et al.^43^ | the Association of Anesthetists and the British Pain Society | 2024 | Expert consensus | Peri-operative pain management in adults |
| Makkad et al.^44^ | the Society of Cardiovascular Anesthesiologists | 2023 | Expert consensus | Practice advisory for preoperative and intraoperative pain management of thoracic surgical patients |
| Zhang et al.^45^ | CNKI | 2021 | Expert consensus | Perioperative goal-oriented whole-process analgesia management |
| Chinese Society of cardiothoracic Anesthesia Day Surgery anesthesia branch^46^ | CNKI | 2021 | Expert consensus | Pediatric accelerated rehabilitation surgical anesthesia |
| Wan et al.^47^ | CNKI | 2020 | Expert consensus | Chinese expert consensus on the application of pain assessment scales |
| Thoracoscopic Surgery Group, Thoracic and Cardiovascular Surgery Society, Chinese Medical Association^48^ | CNKI | 2018 | Expert consensus | Expert consensus on perioperative pain management in thoracic surgery in China |
| Mayer H^56^ | UpToDate | 2024 | Clinical decision | Pectus excavatum: treatment |
| Zacha et al.^49^ | PubMed | 2024 | Systematic review | Cryoanalgesia as the essential element of enhanced recovery after surgery (eras) in children undergoing thoracic surgery |
| Chen et al.^50^ | PubMed | 2023 | Systematic review | Continuous nerve block versus thoracic epidural analgesia for post-operative pain of pectus excavatum repair |
| Eldredge et al.^51^ | PubMed | 2023 | Systematic review | Intercostal nerve cryoablation therapy for the repair of pectus excavatum |
| Feray et al.^52^ | PubMed | 2022 | Systematic review | Procedure-specific postoperative pain management recommendations for video-assisted thoracoscopic surgery |
| Heo et al.^53^ | PubMed | 2021 | Systematic review | Epidural analgesia versus intravenous analgesia after minimally invasive repair of pectus excavatum in pediatric patients |
| Daemen et al.^54^ | PubMed | 2020 | Systematic review | Intercostal nerve cryoablation versus thoracic epidural for postoperative analgesia following pectus excavatum repair |
| Archer et al.^55^ | PubMed | 2020 | Systematic review | Postoperative pain control following minimally invasive correction of pectus excavatum in pediatric patients |
| Kim et al.^57^ | PubMed | 2024 | RCT | Impact of dexmedetomidine-based opioid-sparing anesthesia on opioid use after minimally invasive repair of pectus excavatum |
| Rim et al.^58^ | PubMed | 2024 | RCT | Cryoanalgesia for pain management following pectus excavatum repair |
| Xu et al.^59^ | PubMed | 2024 | RCT | Erector spinae plane block provided comparable analgesia as thoracic paravertebral block post pediatric Nuss procedure for pectus excavatum |
| Ren et al.^60^ | PubMed | 2024 | RCT | Ultrasound-guided erector spinae plane block versus thoracic epidural block for postoperative analgesia in pediatric Nuss surgery |
| Zhang et al.^61^ | PubMed | 2023 | RCT | Application of multimodal analgesia based on single thoracic epidural block in children undergoing Nuss surgery |
| Fenikowski et al.^62^ | PubMed | 2022 | RCT | The effects of gabapentin on post-operative pain and anxiety, morphine consumption and patient satisfaction in pediatric patients following the Ravitch procedure |
| Ma et al.^63^ | PubMed | 2022 | RCT | Patient-controlled intravenous analgesia with or without ultrasound-guided bilateral intercostal nerve blocks in children undergoing the Nuss procedure |
| Sujka et al.^64^ | PubMed | 2020 | RCT | Epidural versus PCA pain management after pectus excavatum repair |
| Graves et al.^65^ | Citation searching | 2019 | RCT | Intraoperative intercostal nerve cryoablation during the Nuss procedure reduces length of stay and opioid requirement |
| Luo et al.^66^ | PubMed | 2017 | RCT | Comparison of ultrasonography-guided bilateral intercostal nerve blocks and conventional patient-controlled intravenous analgesia for pain control after the Nuss procedure in children |
| Qi et al.^67^ | PubMed | 2014 | RCT | A prospective randomized observer-blinded study to assess postoperative analgesia provided by an ultrasound-guided bilateral thoracic paravertebral block for children undergoing the Nuss procedure |
| Wharton et al.^68^ | PubMed | 2024 | Cohort study | Enhanced recovery after surgery 2.0: optimizing pain management in Nuss procedure: cryoablation and nerve block strategies for reduced opioid use |
| Linton et al.^69^ | PubMed | 2024 | Cohort study | Intercostal nerve cryoablation reduces opioid use and length of stay without increasing adverse events: a retrospective cohort study of 5442 patients undergoing surgical correction of pectus excavatum |
| Cockrell et al.^70^ | Citation searching | 2023 | Cohort study | Implementation of a cryoablation-based pain management protocol for pectus excavatum |
| Lai et al.^71^ | Citation searching | 2023 | Cohort study | Cryoablation in 350 Nuss procedures: evolution of hospital length of stay and opioid use |
| Hegde et al.^72^ | Citation searching | 2023 | Cohort study | Impact of cryoanalgesia use during minimally invasive pectus excavatum repair on hospital days and total hospital costs among pediatric patients |
| Arshad et al.^73^ | Citation searching | 2023 | Cohort study | Multicenter assessment of cryoanalgesia use in minimally invasive repair of pectus excavatum: a 20-center retrospective cohort study |
| Downing et al.^74^ | PubMed | 2023 | Cohort study | Development of a five-point enhanced recovery protocol for pectus excavatum surgery |
| DiFiore et al.^75^ | Citation searching | 2022 | Cohort study | Next day discharge after the Nuss procedure using intercostal nerve cryoablation, intercostal nerve blocks, and a perioperative eras pain protocol |

| **Supplementary Table 2.** The quality evaluation of the included guidelines (n = 6). | | | | | | | | | | |
| --- | --- | --- | --- | --- | --- | --- | --- | --- | --- | --- |
| **Guidelines** | **Standardized scores in various domains (%)** | | | | | |  | **≥60%** | **≥30%** | **Quality grade** |
|  | **Scope**  **and purpose** | **Stakeholder**  **involvement** | **Rigour** | **Clarity** | **Applicability** | **Independence** | **The overall quality** |  |  |  |
| Dunning et al.^37^ | 87.50% | 79.17% | 83.85% | 95.83% | 66.67% | 95.83% | 6.5 | 6 | 6 | A |
| Vittinghoff et al.^38^ | 95.83% | 88.89% | 78.65% | 93.06% | 63.54% | 91.67% | 6 | 6 | 6 | A |
| RCoA^39^ | 93.06% | 79.17% | 84.90% | 95.83% | 64.58% | 87.50% | 5.75 | 6 | 6 | A |
| Xue et al.^40^ | 87.50% | 80.56% | 70.83% | 90.28% | 44.79% | 91.67% | 5.25 | 5 | 6 | B |
| Cravero et al.^41^ | 95.83% | 84.72% | 80.21% | 93.06% | 52.08% | 87.50% | 6 | 5 | 6 | B |
| Chou et al.^42^ | 98.61% | 87.50% | 93.75% | 97.22% | 69.79% | 97.92% | 6.25 | 6 | 6 | A |

| **Supplementary Table 3.** The quality evaluation of expert consensus (n = 6). | | | | | | |
| --- | --- | --- | --- | --- | --- | --- |
| Items | El-Boghdadly et al.^43^ | Makkad et al.^44^ | Zhang et al.^45^ | Zhu et al.^46^ | Wan et al.^47^ | Liu et al.^48^ |
| 1. Is the source of the opinion clearly identified? | Yes | Yes | Yes | Yes | Yes | Yes |
| 1. Does the source of opinion have standing in the field of expertise? | Yes | Yes | Yes | Yes | Yes | Yes |
| 1. Are the interests of the relevant population the central focus of the opinion? | Yes | Yes | Yes | Yes | Yes | Yes |
| 1. Does the opinion demonstrate a logically defended argument to support the conclusions drawn? | Yes | Yes | Yes | Yes | Yes | Yes |
| 1. Is there reference to the extant literature? | Yes | Yes | Yes | Yes | Yes | Yes |
| 1. Is any incongruence with the literature/sources logically defended? | Yes | Unclear | Unclear | Yes | Unclear | Unclear |

| **Supplementary Table 4.** The quality evaluation of systematic reviews (n = 7). | | | | | | | |
| --- | --- | --- | --- | --- | --- | --- | --- |
| Items | Zacha et al.^49^ | Chen et al.^[50]^ | Eldredge et al.^51^ | Feray et al.^52^ | Heo et al.^53^ | Daemen et al.^54^ | Archer et al.^55^ |
| 1. Is the review question clearly and explicitly stated? | Yes | Yes | Yes | Yes | Yes | Yes | Yes |
| 1. Were the inclusion criteria appropriate for the review question? | Yes | Yes | Yes | Yes | Yes | Yes | Yes |
| 1. Was the search strategy appropriate? | Yes | Yes | Yes | Yes | Yes | Yes | Yes |
| 1. Were the sources and resources used to search for studies adequate? | Yes | Yes | Partial yes | Yes | Yes | Yes | Yes |
| 1. Were the criteria for appraising studies appropriate? | Yes | Yes | Yes | Yes | Yes | Yes | Yes |
| 1. Was critical appraisal conducted by two or more reviewers independently? | Yes | Yes | Yes | Yes | Yes | Yes | Yes |
| 1. Were there methods to minimize errors in data extraction? | No | Yes | Unclear | Yes | Yes | Yes | No |
| 1. Were the methods used to combine studies appropriate? | Not applicable | Yes | Not applicable | Yes | Yes | Yes | Partial yes |
| 1. Was the likelihood of publication bias assessed? | Not applicable | Yes | Unclear | Not applicable | Yes | Yes | Not applicable |
| 1. Were recommendations for policy and/or practice supported by the reported data? | Yes | Yes | Partial yes | Yes | Yes | Yes | Unclear |
| 1. Were the specific directives for new research appropriate? | Yes | Yes | Partial yes | Yes | Yes | Yes | Yes |

| **Supplementary Table 5.** The quality evaluation of randomized controlled trials (n = 11). | | | | | | | | | | | | |
| --- | --- | --- | --- | --- | --- | --- | --- | --- | --- | --- | --- | --- |
| **Items** | Kim et al.^57^ | Rim et al.^58^ | Xu et al.^59^ | Ren et al.^60^ | Zhang et al.^61^ | Fenikowski et al.^62^ | Ma et al.^63^ | Sujka et al.^64^ | Graves et al.^65^ | Luo et al.^66^ | Qi et al.^67^ |  |
| ***Bias related to selection and allocation*** | | | | | | | | | | | |  |
| 1. Was true randomization used for assignment of participants to treatment groups? | Yes | Yes | Yes | Yes | Yes | Yes | Yes | Yes | Yes | Yes | Yes |  |
| 1. Was allocation to treatment groups concealed? | Unclear | Yes | Yes | Yes | Unclear | Yes | Yes | Yes | Yes | Yes | Yes |  |
| 1. Were treatment groups similar at the baseline? | Yes | Yes | Yes | Yes | Yes | Yes | Yes | Yes | Yes | Yes | Yes |  |
| *Bias related to administration of intervention/exposure* | | | | | | | | | | | |  |
| 1. Were participants blind to treatment assignment? | Yes | Yes | Yes | Yes | Unclear | Yes | Yes | Unclear | Yes | Yes | Yes |  |
| 1. Were those delivering the treatment blind to treatment assignment? | No | No | Yes | Yes | Unclear | Yes | Yes | Unclear | No | Yes | Yes |  |
| 1. Were treatment groups treated identically other than the intervention of interest? | Yes | Yes | Yes | Yes | Yes | Yes | Yes | Yes | Yes | Yes | Yes |  |
| ***Bias related to assessment, detection and measurement of the outcome*** | | | | | | | | | | | |  |
| 1. Were outcome assessors blind to treatment assignment? | Yes | Yes | Yes | Yes | Unclear | Yes | Yes | Unclear | Unclear | Yes | Yes |  |
| 1. Were outcomes measured in the same way for treatment groups? | Yes | Yes | Yes | Yes | Yes | Yes | Yes | Yes | Yes | Yes | Yes |  |
| 1. Were outcomes measured in a reliable way | Yes | Yes | Yes | Yes | Yes | Yes | Yes | Yes | Yes | Yes | Yes |  |
| ***Bias related to participant retention*** | | | | | | | | | | | |  |
| 1. Was follow up complete and if not, were differences between groups in terms of their follow up adequately described and analyzed? | Yes | Yes | Yes | Yes | Unclear | Yes | Yes | Yes | Yes | Yes | Yes |  |
| ***Statistical Conclusion Validity*** | | | | | | | | | | | |  |
| 1. Were participants analysed in the groups to which they were randomized? | Yes | Yes | Yes | Yes | Unclear | Yes | Yes | Yes | Yes | Yes | Yes |  |
| 1. Was appropriate statistical analysis used? | Yes | Yes | Yes | Yes | Yes | Yes | Yes | Yes | Yes | Yes | Yes |  |
| 1. Was the trial design appropriate and any deviations from the standard RCT design (individual randomization, parallel groups) accounted for in the conduct and analysis of the trial? | Yes | Yes | Yes | Yes | Yes | Yes | Yes | Yes | Yes | Yes | Yes |  |

| **Supplementary Table 6.** The quality evaluation of cohort studies (n = 8). | | | | | | | | |  |
| --- | --- | --- | --- | --- | --- | --- | --- | --- | --- |
| **Items** | Wharton et al.^68^ | Linton et al.^69^ | Cockrell et al.^70^ | Lai et al.^71^ | Hegde et al.^72^ | Arshad et al.^73^ | Downing et al.^74^ | DiFiore et al.^75^ | |
| 1. Were the two groups similar and recruited from the same population? | Yes | Yes | Yes | Yes | Yes | Yes | Yes | Yes | |
| 1. Were the exposures measured similarly to assign people to both exposed and unexposed groups? | Yes | Yes | Yes | Yes | Yes | Yes | Yes | Yes | |
| 1. Was the exposure measured in a valid and reliable way? | Yes | Yes | Yes | Yes | Yes | Yes | Yes | Yes | |
| 1. Were confounding factors identified? | Yes | Yes | Yes | Yes | Yes | Yes | Yes | Yes | |
| 1. Were strategies to deal with confounding factors stated? | Yes | Yes | Yes | Yes | Yes | Yes | Yes | Yes | |
| 1. Were the groups/participants free of the outcome at the start of the study (or at the moment of exposure)? | Yes | Yes | Yes | Yes | Yes | Yes | Yes | Yes | |
| 1. Were the outcomes measured in a valid and reliable way? | Yes | Yes | Yes | Yes | Yes | Yes | Yes | Yes | |
| 1. Was the follow up time reported and sufficient to be long enough for outcomes to occur? | Yes | Yes | Yes | Yes | Yes | Yes | Yes | Yes | |
| 1. Was follow up complete, and if not, were the reasons to loss to follow up described and explored? | Yes | Yes | Yes | Yes | Yes | Yes | Yes | Yes | |
| 1. Were strategies to address incomplete follow up utilized? | Yes | Yes | Not applicable | Yes | Yes | Yes | Yes | Yes | |
| 1. Was appropriate statistical analysis used? | Yes | Yes | Yes | Yes | Yes | Yes | Yes | Yes | |
